# Development of a loop-mediated isothermal amplification technique and comparison with quantitative real-time PCR for the rapid visual detection of canine neosporosis

**DOI:** 10.1186/s13071-017-2330-2

**Published:** 2017-08-23

**Authors:** Aongart Mahittikorn, Nipa Thammasonthijarern, Amonrattana Roobthaisong, Ruenruetai Udonsom, Supaluk Popruk, Sukhontha Siri, Hirotake Mori, Yaowalark Sukthana

**Affiliations:** 1 Department of Protozoology, Faculty of Tropical Medicine, Mahidol University, Bangkok, Thailand; 2 Department of Tropical Pediatrics, Faculty of Tropical Medicine, Mahidol University, Bangkok, Thailand; 3 Section of Bacterial Infections, Thailand-Japan Research Collaboration Center on Emerging and Re-emerging Infections, Nonthaburi, Thailand; 4 Department of Epidemiology, Faculty of Public Health, Mahidol University, Bangkok, Thailand

**Keywords:** *Neospora caninum*, Lamp, Quantitative real-time PCR, Dog, Thailand

## Abstract

**Background:**

Dogs are the definitive hosts of *Neospora caninum* and play an important role in the transmission of the parasite. Despite the high sensitivity of existing molecular tools such as quantitative real-time PCR (qPCR), these techniques are not suitable for use in many countries because of equipment costs and difficulties in implementing them for field diagnostics. Therefore, we developed a simplified technique, loop-mediated isothermal amplification (LAMP), for the rapid visual detection of *N. caninum*.

**Methods:**

LAMP specificity was evaluated using a panel containing DNA from a range of different organisms. Sensitivity was evaluated by preparing 10-fold serial dilutions of *N. caninum* tachyzoites and comparing the results with those obtained using qPCR. Assessment of the LAMP results was determined by recognition of a colour change after amplification. The usefulness of the LAMP assay in the field was tested on 396 blood and 115 faecal samples from dogs, and one placenta from a heifer collected in Lopburi, Nakhon Pathom, Sa Kaeo, and Ratchaburi provinces, Thailand.

**Results:**

Specificity of the LAMP technique was shown by its inability to amplify DNA from non-target pathogens or healthy dogs. The detection limit was the equivalent of one genome for both LAMP and qPCR. LAMP and qPCR detected positive *N. caninum* infection in 15 of 396 (3.8%) blood samples; LAMP detected 9/115 (7.8%) positive faecal samples, while qPCR detected 5/115 (4.3%) positive faecal samples. The placental tissue was shown to be positive by both techniques. Agreement between LAMP and qPCR was perfect in blood samples (kappa value, 1.00) and substantial in faecal samples (kappa value, 0.697).

**Conclusions:**

This is the first known LAMP assay developed for the amplification of *N. caninum*. The technique effectively and rapidly detected the parasite with high sensitivity and specificity and was cost-effective. This assay could be used in the field to confirm the diagnosis of canine or bovine neosporosis.

**Electronic supplementary material:**

The online version of this article (doi:10.1186/s13071-017-2330-2) contains supplementary material, which is available to authorized users.

## Background

*Neospora caninum* is an obligate intracellular protozoan that causes neosporosis in dogs and cattle worldwide. Although the structure and life-cycle of *N. caninum* are similar to those of *Toxoplasma gondii*, canids are the only definitive hosts of *N. caninum*, whereas felids are the only definitive host of *T. gondii* [1]. The infection has also been reported in horses, sheep, deer, and goats [2]. Neosporosis is a major cause of abortion and stillbirth in both dairy and beef cattle and is occasionally a problem in a livestock operation. Infected cattle may abort several times because of chronic neosporosis, and any offspring born alive are likely to be themselves infected and have a higher risk of future abortions [3]. Therefore, neosporosis is considered a veterinary health problem that has an economic impact on the livestock industry through losses in beef and milk production [4].

The transmission of *N. caninum* occurs from the ingestion of water or food contaminated with sporulated oocysts or tissue cysts (horizontal transmission), or transplacental infection (vertical transmission). Major modes of transmission in dogs include direct contact with cattle in rural areas enabling the ingestion of infected foetal material, the ingestion of infected intermediate hosts, e.g. rodents, birds, and other animals, and the direct ingestion of oocysts [5]. Because dogs are used on farms as herding or livestock guardians and can shed oocysts when they are infected, it is important for control programs to identify and remove these animals from breeding herds [1].

The diagnosis of canine neosporosis is based on combining appropriate clinical signs and serology such as immunofluorescent antibody tests and enzyme-linked immunosorbent assays (ELISAs) [6]. However, these assays may be limited by false-negative results in early or chronic cases of infection [7]. Moreover, standardised tests are not available, so it is not possible to compare results between different laboratories [2]. The definitive standard for identifying infection is the recognition of tissue cysts on histological examination, the discovery of faecal oocysts, or identification of the parasite by immunohistochemical staining [8]. Light microscopy alone is not enough to identify *N. caninum* oocysts in dog faeces as it is difficult to distinguish *N. caninum* oocysts from those of other canine coccidian parasites such as *Hammondia heydorni*, or from other contaminating oocysts such as *H. hammondi* and *T. gondii* [2]. Immunohistochemical examination, which would aid differentiation, is rarely performed [9]. Moreover, only a few reports have identified *N. caninum* oocysts in naturally-infected dogs [10–13]. Quantitative real-time PCR (qPCR) has also been developed to detect and quantify *N. caninum* DNA in mice [14], rats [15], cattle [16–19], horses [20] and dogs [7, 21]. Although this is highly sensitive and specific, in general, it can only be used in specialised laboratories. It is not applicable to field situations due to a variety of issues, including the high costs and the need for well-trained personnel for DNA isolation, preparation of reaction mixture, etc. [22].

During the last decade, loop-mediated isothermal amplification (LAMP) has been established as a simple, rapid, and cost-effective technique for the detection of pathogens. It offers high sensitivity and specificity even when little target DNA is available [23, 24]. Therefore, the present study aimed to develop LAMP for detecting *N. caninum* infection in dogs in the field. The technique was validated and evaluated using blood, and faecal samples from dogs and the prevalence of canine neosporosis in Thailand was investigated.

## Methods

### Organisms used in this study

*Neospora caninum* strain K9WA, originally isolated from a naturally infected dog in Western Australia, was used as reference DNA. *Neospora caninum* tachyzoites were maintained by serial passage in Vero cell monolayers, in Dulbecco’s modified Eagle’s medium (Hyclone Laboratories, Logan, UT, USA) containing 10% foetal bovine serum (Sigma-Aldrich, St. Louis, MO, USA), as previously described [25]. The number of tachyzoites in the suspension was determined using a haemocytometer, and the suspension was serially diluted 10-fold in sterile PBS as required. DNA was extracted from *T. gondii*, *Cryptosporidium parvum*, *Cyclospora cayetanensis*, *Giardia duodenalis*, *Entamoeba histolytica*, *Entamoeba coli*, *Blastocystis*, *Enterocytozoon bieneusi*, *Naegleria fowleri*, *Acanthamoeba* spp. and *Escherichia coli* stocks in the Faculty of Tropical Medicine (Mahidol University) with a QIAamp DNA Mini Kit (Qiagen, Hilden, Germany) and used for specificity testing.

### *Neospora caninum* LAMP and qPCR primers

LAMP primers were designed from the repeated *Neospora*-specific Nc5 sequence (GenBank: X84238), shown in previous studies to be highly specific for *N. caninum* [7, 26]. Six oligonucleotide primers, consisting of the outer forward primer (F3), outer backward primer (B3), forward inner primer (FIP), backward inner primer (BIP), loop forward (FL), and loop backward (BL), were selected using LAMP Primer designing software PrimerExplorer ver. 4 (http://primerexplorer.jp/elamp4.0.0/index.html). A Basic Local Alignment Search Tool (BLAST) search (http://blast.ncbi.nlm.nih.gov/Blast.cgi) was performed to confirm primer specificity. To make a comparative analysis of LAMP and qPCR, published primers (561 U20 and 806 L20) for qPCR that also targeted the *Neospora*-specific Nc5 sequence were used [7]. All primers were synthesised using High-Affinity Purification from Bio Basic Inc. (Markham, Ontario, Canada). Table 1 provides information about names, sequences and lengths of the primers.

**Table 1:** Primer sets designed for LAMP and qPCR based on the *Neospora*-specific Nc5 gene

Purpose	Primer name	Nucleotide sequence (5′-3′)	Source
LAMP assay	FIP (F1c + F2)	ACAGCCAAACACAACCCGACTC-CATGAGGCCGGAGAATGAGA	This study
	BIP (B1c + B2)	AAGGACAGGGTTGGGTATCGC-GATGCCGCTCCTGAAGTC	This study
	LF	GAAGGAAGACACCTGGAAATCG	This study
	LB	GGAGCTGGGTTGCTGTGCTC	This study
	F3	GGCTTCATGCGAGGTCTC	This study
	B3	TCAGTGAGCGATGTCCTCC	This study
qPCR	561 U20	GGGAGTTGGTAGCGGTGAGA	[7]
	806 L20	GCCTCCCAATGCGAACGAAA	[7]

### LAMP and qPCR

**Fig. 1:**
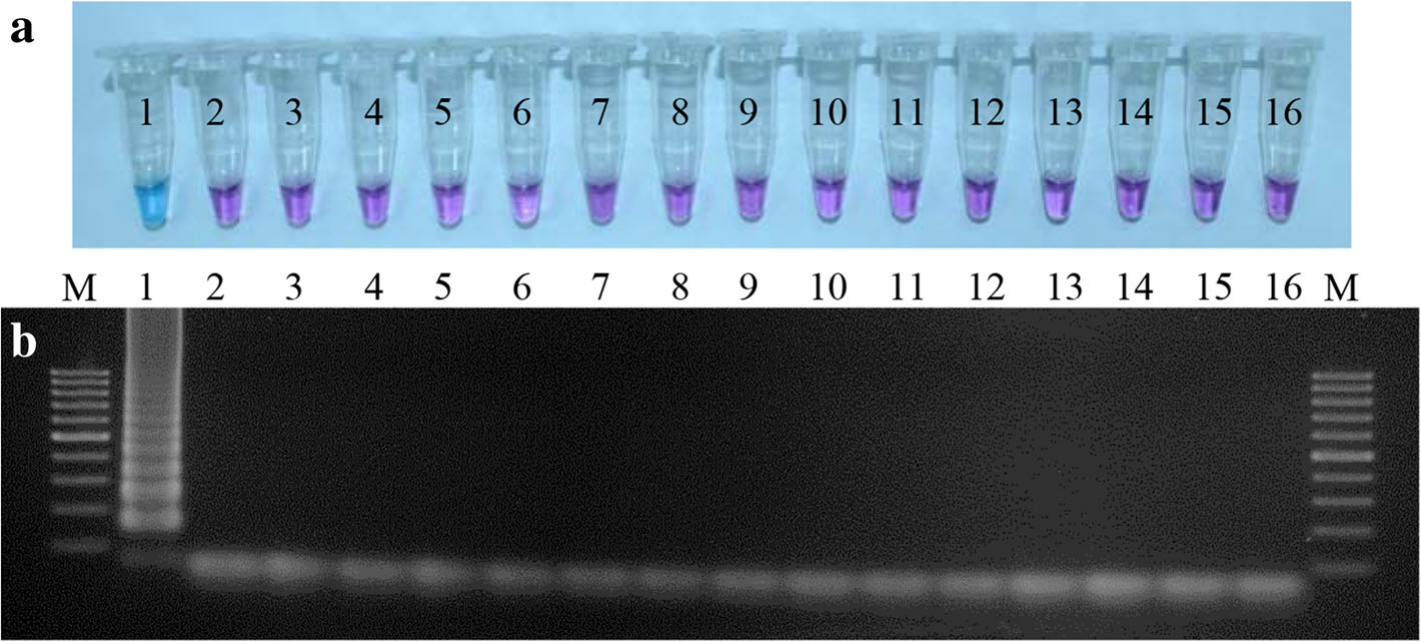
Specificities of the LAMP assay for the detection of *N. caninum*. **a** Specificity of the LAMP assay using HNB (the light blue colour indicates a positive sample). **b** Confirmation of the LAMP results using agarose gel (2%) electrophoresis. Lane M: 100 bp DNA ladder; Lane 1: *N. caninum*; Lane 2: *T. gondii*; Lane 3: *C. parvum*; Lane 4: *C. cayetanensis*; Lane 5: *G. duodenalis*; Lane 6: *E. histolytica*; Lane 7: *Entamoeba coli*; Lane 8: *Blastocystis*; Lane 9: *E. bieneusi*; Lane 10: *N. fowleri*; Lane 11: *Acanthamoeba* spp.; Lane 12: *Escherichia coli*; Lane 13: blood from healthy heifer; Lane 14: blood from healthy dog; Lane 15: faeces from healthy dog; Lane 16: negative control (sterile water)

LAMP reactions were performed at a range of temperatures (60, 61, 62, 63, 64 and 65 °C), reaction times (45, 60, 75, 90, 105 and 120 min), and concentrations of hydroxy naphthol blue (HNB) (80, 100, 120, 140 and 160 μM) to identify optimal conditions. Results were determined by observation, and considered to be positive following a colour change of the solution from violet to light blue; a negative result remained violet (Fig. 1a). A non-template negative control of sterile water and a positive control of DNA extracted from 100 *N. caninum* tachyzoites were included for each LAMP run. All reactions were performed in duplicate, and 2 μl aliquots of LAMP products were run on agarose gel electrophoresis to confirm results. Optimal conditions were identified as amplification at 64 °C for 60 min, with a final concentration of 80 μM HNB. Thereafter, LAMP assays were carried out in 25 μl reaction containing 1.6 μM each of FIP and BIP, 0.2 μM each of F3 and B3, 0.8 μM each of LF and LB, 1.4 mM of each dNTP (Thermo Scientific, Waltham, MA, USA), 0.8 M betaine (Sigma-Aldrich), 8 U of the large fragment of *Bst* DNA polymerase (New England BioLabs, Ipswich, MA, USA), 1× of supplied ThermoPol buffer, 8 mM MgSO_4_ (New England Biolabs), and 80 μM HNB (Sigma-Aldrich) with 2 μl of extracted DNA as a template. Amplification was conducted at 64 °C for 60 min and stopped by incubation at 80 °C for 5 min.

Quantitative real-time PCR was performed on a Rotor-Gene 6000 real-time PCR cycler (Qiagen). Reactions were performed in 20 μl mixtures containing 2 μl of DNA template, 0.5 μM each of 561 U20 and 806 L20 primer, and 1× SsoFast EvaGreen Supermix (Bio-Rad, Hercules, CA, USA) with the following conditions: 98 °C for 2 min followed by 50 cycles of 98 °C for 5 s and 60 °C for 20 s. Amplification specificity was checked by performing a melting curve analysis (from 69 °C to 95 °C).

### Specificity and detection limit testing of LAMP and qPCR

To assess the specificity of LAMP and qPCR assays, we tested DNA templates isolated from the organisms listed earlier, as well as DNA extracted from healthy heifer whole blood, healthy dog whole blood, and healthy dog faeces. The healthy heifer whole blood was collected from a clinically normal heifer that found to be seronegative to *N. caninum* in our previous study [25]. The heifer was maintained in a separate pen, and fed standard hay and commercial cattle concentrate during the entire experiment. The healthy dog blood and faecal samples were collected from a dog with no history or clinical signs of neosporosis. The healthy dog had never been outside the owner’s house and was fed only commercially prepared food. The specificity test was repeated twice using good laboratory practices such as including a positive and negative control for all experiments.

The detection limit of both assays was evaluated by performing 10-fold serial dilutions of purified genomic *N. caninum* as described by Ghalmi [7]. Briefly, genomic DNA from 10^7^ *N. caninum* tachyzoites was extracted using a QIAamp DNA Mini Kit and resuspended in 200 μl TE buffer to a concentration of 5 × 10^4^ equivalent genome per μl. Each assay was performed on 2 μl of a 10-fold serial dilution of this suspension. According to this preparation, the reactions contained the equivalent of 10^5^, 10^4^, 10^3^, 10^2^, 10^1^ and 1 genomes, respectively. Detection limit tests were conducted in triplicate, and the last dilution when all three samples tested positive was considered the detection limit.

### Field collection of dog samples from cattle farms for LAMP/qPCR analysis

To verify if the developed LAMP assay could detect *N. caninum*, 396 ethylenediaminetetraacetic acid (EDTA)-blood samples and 115 faecal samples were collected from dogs at 105 dairy cattle farms in Thailand (Mueang Nakhon Pathom district, Nakhon Pathom Province; Photharam district and Ban Pong district, Ratchaburi Province; Phatthana Nikhom district, Lopburi Province; Watthana Nakhon district, Mueang Sa Kaeo district, and Wang Sombun district, Sa Kaeo Province) between April and July 2012 (see Additional file 1: Figure S1). Because of the difficulty in obtaining faeces from the rectum, we were only able to collect paired blood and faecal samples from 115 dogs. Therefore, only blood samples were collected from the remaining 281 dogs. Approximately 3 ml of blood was collected from the cephalic vein into EDTA tubes by experienced veterinarians from both male and female dogs of different breeds and various ages after obtaining permission from their owners. Samples were kept on ice during transportation and stored at -20 °C until DNA extraction. At the time of sample collection, none of the dogs presented with symptoms compatible with neosporosis, including limb paresis or ulcerous cutaneous lesions. During the time of sample collection at Ratchaburi Province, a heifer was aborting her calf, so her placenta was collected for analysis. DNA was extracted from 200 μl blood or 25 mg placental tissue using the QIAamp DNA Mini Kit according to the manufacturer’s instructions. About 200 mg of each faecal sample was subjected to DNA extraction based on the method used for *Cryptosporidium* oocysts and using the QIAamp DNA Stool Mini Kit (Qiagen) [27, 28]. The protocol involved a pre-incubation step in which the faecal samples were resuspended in ATL buffer and subjected to 5 cycles of freezing at -83 °C for 10 min and thawing at 56 °C for 5 min. The manufacturer-recommended protocol was followed for the remainder of the DNA extraction. LAMP and qPCR assays were performed in triplicate on all samples to compare the results. Reactions without DNA served as negative controls.

To confirm the fidelity of amplification, some LAMP positive products were cross-validated by sequencing [29] with modifications. For this, 1 μl of the LAMP product was included in a PCR with two outer primers, F3 and B3. This was then visualised using 2% agarose gel electrophoresis. An expected 189 bp fragment was extracted, cloned in the Zero Blunt TOPO Cloning Kit (Invitrogen, Carlsbad, CA, USA) and transformed into chemically competent *Escherichia coli* DH5α according to the manufacturer’s recommendations. Plasmid DNA was purified using the QIAprep Spin Miniprep Kit (Qiagen). Insert fragments were amplified using primers M13F and M13R and sequenced using the BigDye Terminator V3.1 Cycle Sequencing Kit (Applied Biosystems, Foster City, CA, USA). Resulting sequences were aligned, and BLAST searched.

### Statistical analysis

The Chi-square (*χ* ^2^) test was used to compare the prevalence of *N. caninum* infection among four provinces. McNemar’s test was used to verify the occurrence of significant differences between the obtained results (positive and negative) by LAMP and qPCR. The degree of agreement between these two techniques was estimated by calculating the Cohen’s kappa value [30]. The values of the kappa coefficients were interpreted according to the criteria of Viera & Garrett [31]. All analyses were conducted using SPSS for Windows 18.0 software (SPSS Inc., Chicago, IL, USA). Statistical significance was defined as *P* < 0.05.

## Results

### Specificity of the LAMP assay

Assay specificity was determined by testing DNA derived from known concentrations of *N. caninum* DNA, as well as that from the other organisms listed, and dog host DNA. Only *N. caninum* DNA could be amplified and detected with the naked eye by observing the colour change of the solution from violet to light blue (Fig. 1a). The LAMP products also exhibited a ladder-like pattern on the gel confirming that the LAMP primers were specific (Fig. 1b). The specificity test for qPCR similarly showed that no amplification occurred in samples containing DNA other than that of *N. caninum* (data not shown).

### The lower limit of detection in LAMP and qPCR assays for the detection of *N. caninum* DNA

**Fig. 2:**
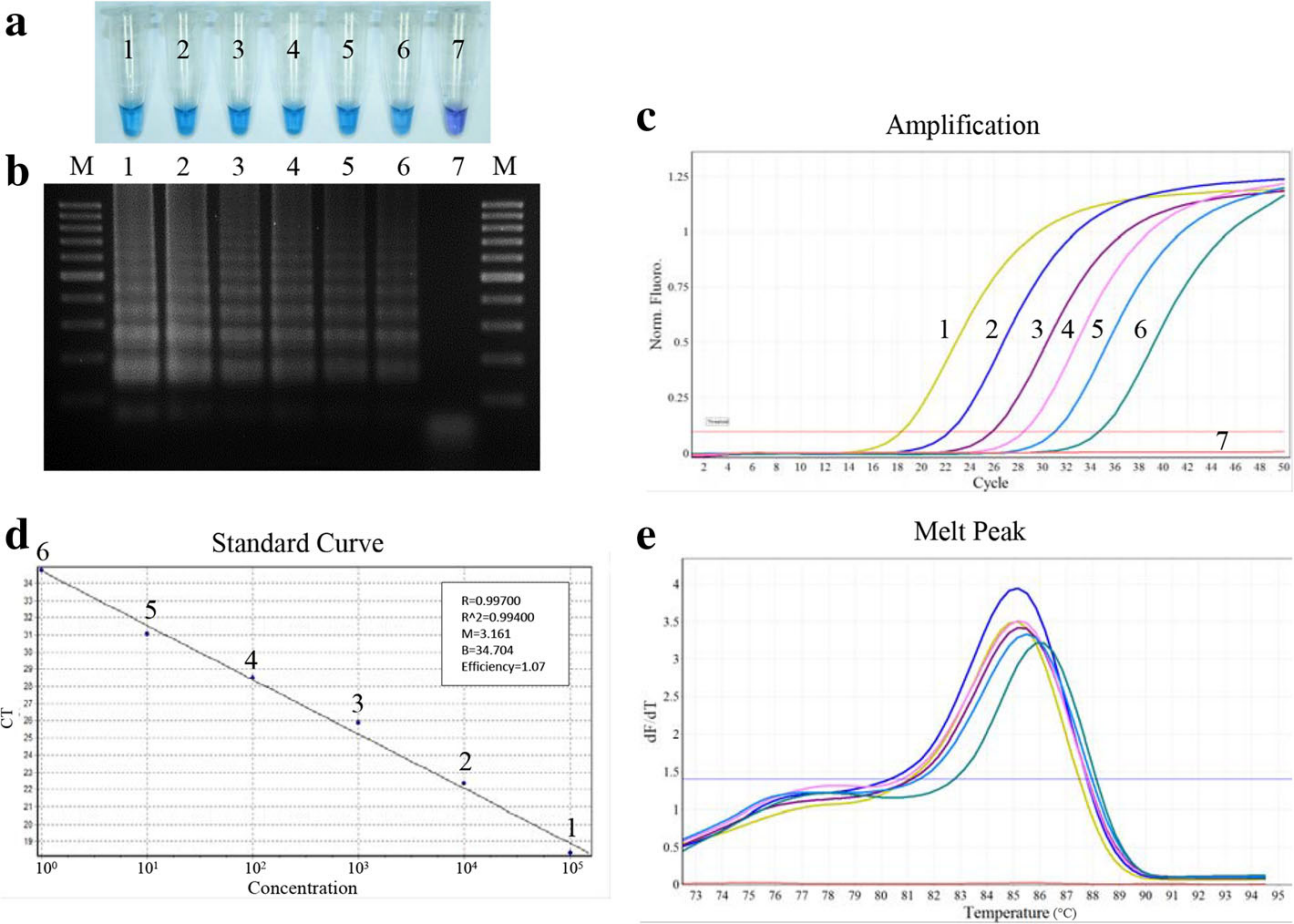
LAMP and qPCR detection limits for 10-fold serial dilutions of *N. caninum* DNA. **a** Results of LAMP analysis. **b** Electrophoresis results of LAMP products. **c** Results of qPCR analysis. **d** *N. caninum* DNA standard curve and amplification efficiency. The crossing point values are plotted against the log of the initial template concentration (equivalent genomes of *N. caninum* DNA). **e** *N. caninum* DNA melting curves. Lane M: 100 bp DNA ladder; Lanes 1–7: 10^5^, 10^4^, 10^3^, 10^2^, 10 and 1 equivalent genomes of *N. caninum* DNA, respectively; Lane 7: negative control (sterile water)

The lower limit of detection was determined using serially diluted *N. caninum* DNA. The concentrations tested for LAMP were positive at all dilutions (Fig. 2a, b). The standard curve generated by qPCR using the 10-fold serial dilutions of DNA was linear over six orders of magnitude (1–10^5^ equivalent genomes) (Fig. 2c, d). The qPCR melting curve analysis monitoring product amplification showed a unique peak at 85.5–86.3 °C (Fig. 2e).

### Applications and comparison of LAMP and qPCR for the detection of *N. caninum*-infected dogs in Thailand

The prevalence of *N. caninum* infection as determined by LAMP was highest in Ratchaburi Province (8.2%), followed by Nakhon Pathom Province (6.7%), Sa Kaeo Province (5.3%), and Lopburi Province (0.6%) (Table 2). Significant differences in prevalence were observed among the four provinces according to both assays (*χ* ^*2*^ = 11.327, *df* = 3, *P* = 0.011 by LAMP, *χ* ^*2*^ = 14.807, *df* = 3, *P* = 0.002 by qPCR).

**Table 2:** Locations, sample types, and infection analysis by LAMP and qPCR

Location and type of samples (no. of samples)	LAMP-positive (%)***	qPCR-positive (%)****
Lopburi Province	1 (0.6)	1 (0.6)
Blood (135)	1 (0.7)	1 (0.7)
Faeces (33)	0 (0)	0 (0)
Nakhon Pathom Province	2 (6.7)	2 (6.7)
Blood (27)	2 (7.4)	2 (7.4)
Faeces (3)	0 (0)	0 (0)
Sa Kaeo Province	7 (5.3)	3 (2.3)
Blood (80)	3 (3.8)	3 (3.8)
Faeces (52)	4 (7.7)	0 (0)
Ratchaburi Province	15 (8.2)	15 (8.2)
Blood (154)	9 (5.8)	9 (5.8)
Faeces (27)	5 (18.5)	5 (18.5)
Placenta (1)	1 (100)	1 (100)
Total blood (396)*	15 (3.8)	15 (3.8)
Total faeces (115)**	9 (7.8)	5 (4.3)
Grand totals (512)	25 (4.6)	21 (3.9)

*P-value for McNemar’s test among blood samples = 1.00

**P-value for McNemar’s test among faecal samples = 0.125

***P-value for Chi-square test for prevalence among the four provinces by LAMP = 0.011

****P-value for Chi-square test for prevalence among the four provinces by qPCR = 0.002

Both LAMP and qPCR detected positive *N. caninum* infection in 15 of 396 (3.8%) dog blood samples (see Additional file 2: Table S1, Additional file 3: Figure S2). No discrepancies were identified because both tests identified the same dogs as having a positive infection. However, LAMP showed that 9 of 115 (7.8%) dog faecal samples were positive for infection while qPCR only detected infection in 5 of 115 faecal samples (4.3%), all of which were also shown to be positive by LAMP. Only one dog was found to be positive by both techniques in blood and faeces (see Additional file 2: Table S1; Sample no. 99). When plotted on a standard curve consisting of serially diluted DNA from *N. caninum*, quantification cycle (C_q_) values were equivalent to a range of 25.51 to 32.07 cycles, corresponding to 813 to 7 genome equivalents for all positive blood samples, and 31.25 to 32.85 cycles, corresponding to 12 to 4 genome equivalents for all positive faecal samples. All discrepant results for faecal samples derived from Sa Kaeo Province. *N. caninum* DNA was detected in placental tissue of the heifer at a farm in Ratchaburi Province by both LAMP and qPCR assays (C_q_ value of 28.52, corresponding to 91 genome equivalents), which is in accordance with the clinical history of the heifer. The dog showed by both LAMP, and qPCR analysis of blood and faecal samples to be *N. caninum*-positive also resided at this farm. The positive LAMP products from dog samples and the placental tissue were re-amplified by PCR, sequenced and submitted to GenBank (accession nos. MF084988–MF084990). The sequences had the best scores with *N. caninum* (99% homology) which provided a definitive genomic identification of the infections.

No significant differences were identified between the two techniques regarding the positive and negative results. Moreover, 100% concordance (396/396) of positivity and negativity between the two assays was detected in blood samples (McNemar’s test, *P* = 1.00), and 96.5% concordance (111 of 115) was detected in faecal samples (McNemar’s test, *P* = 0.125) as shown in Table 2. The agreement between LAMP and qPCR analysis of blood samples was considered to be perfect with a kappa value of 1.00, and substantial (kappa value = 0.697) for detection in faeces (Table 3).

**Table 3:** Agreement (Kappa) between the detection of *N. caninum* by LAMP and qPCR in blood and faecal samples

qPCR result	LAMP result		Kappa value	*P*-value
	positive	negative		
Blood samples (*n* = 396)				
Positive	15	0	1.00	< 0.001
Negative	0	381		
Faecal samples (*n* = 115)				
Positive	5	0	0.697	< 0.001
Negative	4	106		

## Discussion

Dogs, especially farm dogs, are an important factor in the spread of *N. caninum* infection. They can become infected by ingesting tissues such as livers, lungs, and brains, as well as aborted foetuses and placental material from infected cattle [32]. Following infection, *N. caninum* oocysts are shed in their faeces which contaminate water, pasture, and the environment of cattle farms. Although there are few reports of dogs naturally shedding oocysts, 503,300 oocysts were detected in a dog that was fed with experimentally-infected calf tissues and 820,655 were found in a dog fed naturally-infected buffalo brain [33, 34]. Although infected dogs normally shed oocysts for only a short period, Gondim et al. [35] reported that they might re-shed oocysts after feeding on infected tissue 18–20 months after the primary infection, which might increase the transmission of the parasite to cattle but this remains in doubt.

To prevent significant losses in farm production, the rapid detection and control of *N. caninum* infection in dogs are required and have been an objective for many studies [2]. The implementation of qPCR assays has revealed their major advantages over conventional molecular detection techniques [7, 17, 21]. However, significant dominance in the use of LAMP assays with non-instrumented fluorescent detection is reported. In the present study, the lower limit of LAMP detection was equivalent to that of qPCR (Fig. 2), the per-test consumable costs were similar, but the equipment costs for LAMP carried out using a water bath, or heated block was considerably lower [36–39]. This potentially makes the LAMP technique more suitable for use outside conventional laboratory facilities or in small-scale diagnostic laboratories where established PCR-based methods are too slow, expensive, or complicated for routine use. It could also be beneficial to industry, where the rapid assessment of the parasite infection status in or near farms could be used to make decisions relating to the management of neosporosis. Moreover, the time to complete LAMP and qPCR is similar (within 1.5 h), which is much faster than that of nested or conventional PCR (approximately 2.5–4 h plus the time required for gel electrophoresis) [40].

LAMP has successfully been used in the identification of parasitic infections in dogs, including giardiasis, leishmaniasis, and babesiosis [36–38]. To the best of our knowledge, this is the first LAMP technique to be developed for the diagnosis of neosporosis. It offers high specificity because it uses six primers to recognise six to eight regions on the target DNA for amplification [40]. Although the assay was not tested on parasites phylogenetically related to *N. caninum* such as *Hammondia* and *Sarcocystis* because of the absence of these organisms in our sample repository, we designed LAMP primers based on the unique, repetitive *Neospora*-specific Nc5 sequence which has not been detected in other taxa [7, 23]. We also checked the primer specificity against the GenBank database to ensure that non-specific binding was eliminated. Our results proved that the LAMP primers are highly specific and sensitive for the detection of *N. caninum* DNA (Figs. 1a, 2a). These results also demonstrate that qPCR and LAMP can be used to accurately detect target DNA over a wide range of concentrations with a detection limit equivalent to one genome. With regard to sensitivity level achieved by LAMP in this study, similar results were observed by Ghalmi et al. [7] who reported the same sensitivity of the equivalent of one genome by qPCR systems, while Han et al. [21] demonstrated a lower sensitivity of 38 molecules per microliter by using multiplex qPCR.

The detection of LAMP results is usually determined by observing turbidity with the naked eye or adding intercalating dyes such as SYBR green [36] or propidium iodide [39] to the reaction tube after amplification. However, these methods require a degree of skill or UV light to assess the result. With the use of HNB, LAMP products can be readily detected by visual inspection, thereby reducing the reaction time and enabling the assay to be used for field testing. Additionally, HNB minimises the risk of obtaining false positives through aerosol contamination because it is added before amplification [41]. For these reasons, the LAMP technique developed here appears to be a promising alternative to qPCR for the detection of *N. caninum*.

In Thailand, the seroprevalence of bovine neosporosis in dairy cattle farms ranges from 3 to 47% depending on the type of serological test and cut-off level used, and the areas surveyed [32, 42–44]. Few studies have reported the presence of *N. caninum* in dogs, although 1.2% of dogs near farms in Nakhon Pathom Province were shown to be *N. caninum-*positive by competitive ELISA [42]. In another study carried out in Western Thailand, 7% of dogs were found to be positive for the same test, and 4.4% by an indirect fluorescent antibody test [32]. Although serological antibody testing is the most common method used for dogs that have the past or recent contact with the parasite, it is not correlated with the shedding of oocysts [45]. Moreover, several studies have shown that dogs can shed oocysts without demonstrating seroconversion [11, 33, 45].

Despite the importance of dogs in the epidemiology of the disease, only a few studies have reported the prevalence of *N. caninum* from faecal samples or by using molecular techniques [46]; ours is the first such study in Thailand. Therefore, the prevalence and incidence of *N. caninum* oocyst shedding by dogs remain unclear. We detected *N. caninum* DNA in 3.8% of blood samples by LAMP and qPCR which was lower than that reported from a molecular survey of dogs in Algeria (13%) [47]. However, we observed a perfect agreement between the LAMP and qPCR results for the analysis of blood samples. It is possible that the marked difference in prevalence between these two studies reflects the geographical region because the seroprevalence in Algerian dogs was as high as 22% in stray dogs and 44% in farm dogs [47].

It has previously been shown that *N. caninum* DNA can be amplified from the blood of infected aborted and pregnant cows [17, 48], bulls [49], mice [50] and sheep [51, 52]. Ghalmi et al. [47] also showed that circulating parasites or parasite DNA could be detected in the blood of naturally infected dogs. In the present study, we confirmed that the parasite DNA could be detected in the blood of dogs, indicating that either an acute infection resulted in the circulation of tachyzoites in the blood or that a latent infection released bradyzoites which differentiated into circulating tachyzoites that later formed a new cyst [17, 53]. Information concerning the duration of parasitemia in infected dogs is limited. When toxoplasmosis in cats was assessed by PCR, a cat that was positive in blood would likely have shed oocysts within the previous 35 days [54, 55]. As *N. caninum* and *T. gondii* are very closely related parasites, although faecal examination under the microscope was not performed in the present study, dogs with positive results were also likely to have shed oocysts during the period. However, care must be taken in making generalisations about *N. caninum* based on the biology of *T. gondii* because neosporosis and toxoplasmosis are biologically distinct diseases [2]. Molecular assays are useful for early detection of *N. caninum* in asymptomatic animals [7, 17, 47, 52] because blood seems to provide the transport medium for tachyzoites between body tissues [48]. However, establishing an epidemiological relevance or cause-effect relationship between abortion and *N. caninum* is difficult because it was not known that detected DNA derived from multiplying tachyzoites in blood or free floating DNA, and finding the presence of the parasite DNA does not mean that *N. caninum* caused the abortion [1]. Unfortunately, from 15 positive blood samples, only two faecal samples were collected from the same dogs (see Additional file 2: Table S1; Sample no. 40 and 99) but the sample no. 40 showed ambiguous results between blood and faeces. Therefore, if detection of *N. caninum* DNA in blood corresponds to the detection of DNA in faeces remains unclear. Epidemiological or experimental studies using molecular techniques versus immunological techniques in dog blood with variable aspects should be performed in detail to understand the implication for farm management better.

*Neospora caninum* oocysts are highly resistant to disinfectants and can remain infectious for many months in the environment; however, they are killed by temperatures above 100 °C [56]. Identifying oocysts in environmental samples is undertaken by dairy farms that have experienced an outbreak to prevent neosporosis-associated abortions [1]. Several methods are available to diagnose neosporosis as a cause of abortion. The preferred samples in cases of abortion include one or more aborted foetuses submitted with the placenta and sera from the dam [57]. The positive results obtained in the present study from the placental tissue of an aborted calf prove the applicability of LAMP to many sample types and suggest that it could be used as a definite diagnosis to confirm neosporosis.

In the present study, the prevalence of detected DNA in dog faeces was shown to be 7.8 and 4.3% by LAMP and qPCR, respectively, similar to the previous reports in Ethiopia [10] and Costa Rica [58] but somewhat lower compared to China [59], and higher compared to Iran [11]. The discrepancy of LAMP and qPCR perhaps related the very low levels of DNA in the samples, which may have been beyond the detection limit of qPCR; LAMP is typically one to two orders of magnitude higher in sensitivity than qPCR [60]. Alternatively, the difference in sensitivity may be related to the presence of inhibitors, such as bile salts and plant-based polysaccharides that are known to be found in faecal samples [61, 62].

Theoretically, *Bst* polymerase used in LAMP has a higher tolerance of inhibitors than *Taq* polymerase used in qPCR [63, 64]. This suggests that the sensitivity of LAMP is slightly higher than that of qPCR, which is useful for the direct application of field faecal samples. In this study, most dogs that tested positive for *N. caninum* infection were from farms that allowed them to eat foetal tissues, placentas, or calf carcases. These findings are consistent with reports of high neosporosis prevalence in farm dogs which is linked with the existence of infective materials [47, 58, 65]. Hence, good management practices are important to reduce the risk of transmission from cattle to dogs and vice versa, especially in highly endemic areas. However, the positive coprological LAMP detections may also be due to the possible presence of sexual parasite forms in the infected enteric epithelial cells and included in the samples that DNA was extracted from [66]. An analysis to determine LAMP detection sensitivity, timing, consistency and the stage of *N. caninum* detected compared with traditional methods (microscopy and bioassay) should be explored in future studies.

Despite obvious advantages of the LAMP technique used in this study, future issues remain to be addressed and improved. These include further validation with other tissues such as brain, heart, liver, placenta, and body fluids, as well as samples from the field to prove that this assay can be used to detect *N*. *caninum* infection in cattle rapidly. Secondly, the application of faecal sample preparation and DNA extraction by heat and physical disruption would greatly reduce the need for instruments and the overall cost, making the test simpler to perform in remote areas [62, 67]. Finally, the LAMP assay could be used to monitor amplification in real-time, which could be achieved with a turbidimeter [40, 68]. However, the high amplification efficiency should be taken into account, which could increase the occurrence of carry-over contamination compared with conventional PCR [69–71]. Hence, all precautions to prevent cross-contamination should be carefully observed.

## Conclusion

We have developed the first known LAMP technique to detect *N. caninum* with high specificity and sensitivity. The immediate visual detection of positive results and the use of basic heating devices make this method simple for use in the field. This LAMP assay has been validated in dog samples and has the potential to achieve the rapid detection of *N. caninum*. Examination of both blood and faecal samples would probably have increased the number of positive dogs whose faeces may contain oocysts. Further development, modification, validation, and standardisation will provide an asset for diagnosis, epidemiology, control, and prevention strategies for neosporosis.

## Additional files

Additional file 1: Figure S1. Map showing the sampling areas and the types of samples collected in this study. (TIFF 2909 kb)

Additional file 2: Table S1. Results of all dog positive samples. (PDF 84 kb)

Additional file 3: Figure S2. Pictures of all LAMP positive samples. (TIFF 2172 kb)

## Abbreviations

BLAST: Basic Local Alignment Search Tool
C_q_: Quantification cycle
EDTA: Ethylenediaminetetraacetic acid
ELISA: Enzyme-linked immunosorbent assay
HNB: Hydroxy naphthol blue
LAMP: Loop-mediated isothermal amplification
PBS: Phosphate-buffered saline
qPCR: Quantitative real-time PCR
TE: Tris-EDTA
*χ*^2^: Chi-square test
