# Supplementary figures and images for: Development of a loop-mediated isothermal amplification technique and comparison with quantitative real-time PCR for the rapid visual detection of canine neosporosis
Source: Parasit Vectors. 2017 Aug 23;10:394. doi: 10.1186/s13071-017-2330-2 (PMC5569544; doi:10.1186/s13071-017-2330-2)

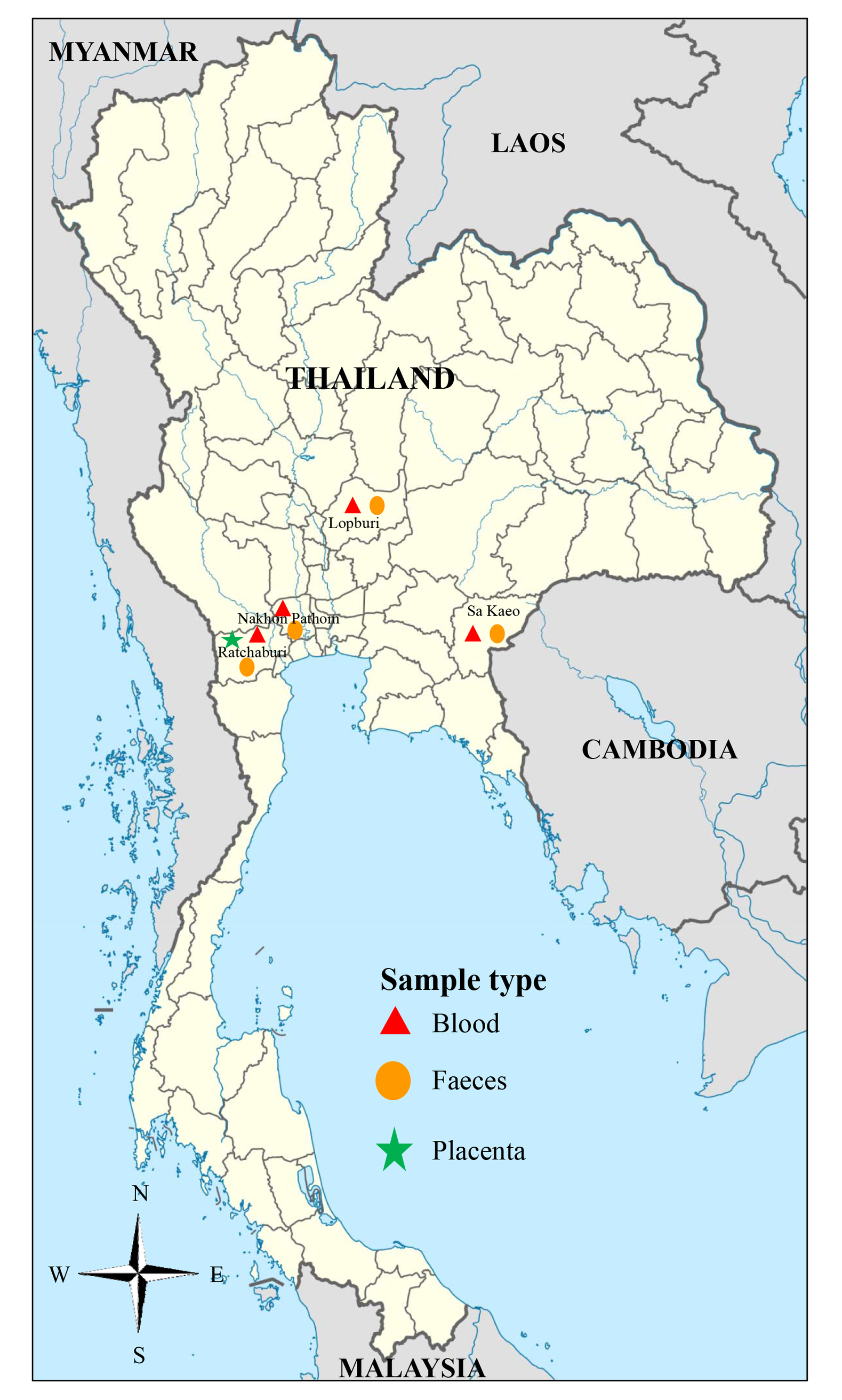

Supplement: Supplementary file 1 — Map showing the sampling areas and the types of samples collected in this study. (TIFF 2909 kb) [file 13071_2017_2330_MOESM1_ESM.tif]

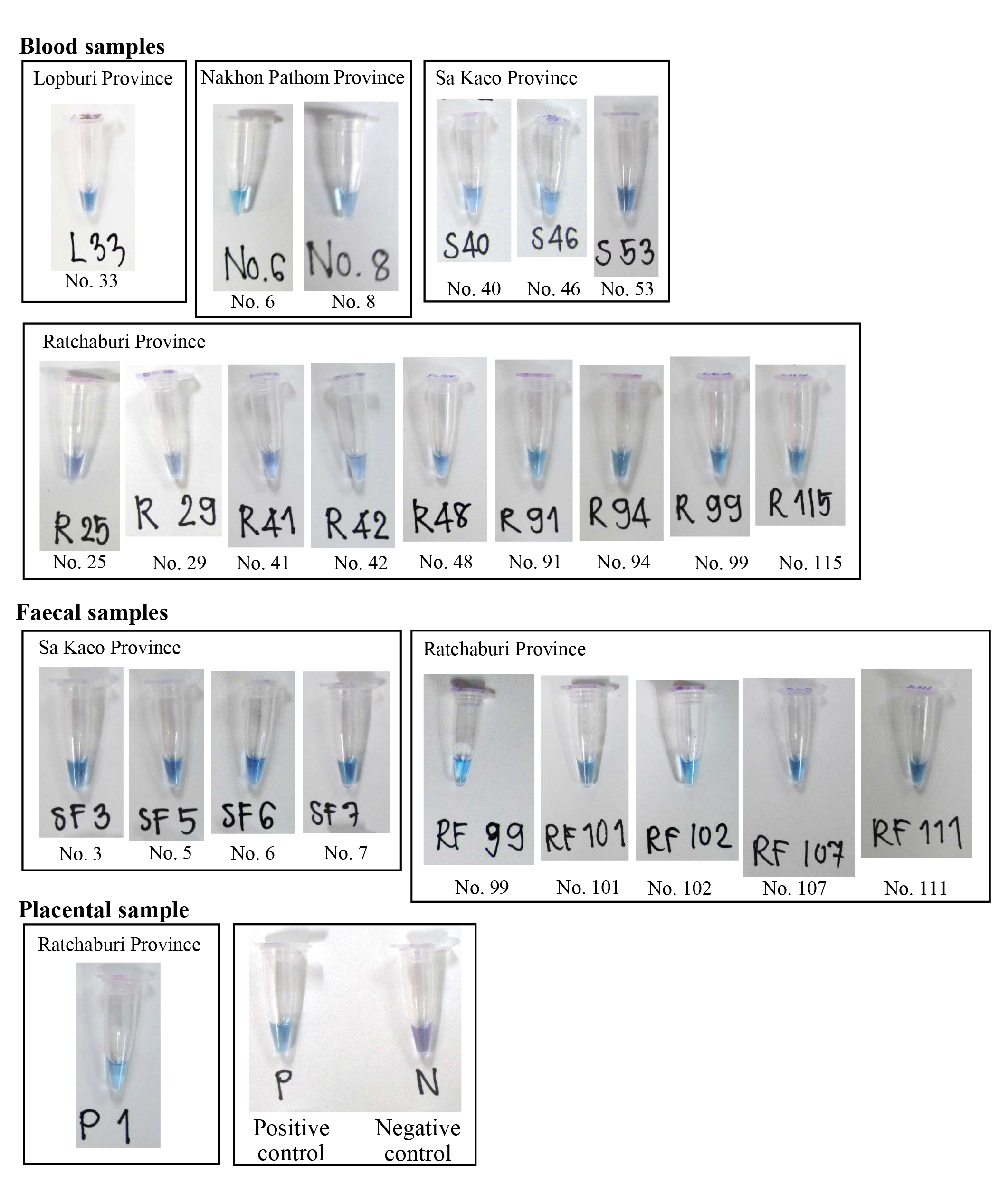

Supplement: Supplementary file 3 — Pictures of all LAMP positive samples. (TIFF 2172 kb) [file 13071_2017_2330_MOESM3_ESM.tif]
