# Supplementary material for: Development of a loop-mediated isothermal amplification technique and comparison with quantitative real-time PCR for the rapid visual detection of canine neosporosis
Source: Parasit Vectors. 2017 Aug 23;10:394. doi: 10.1186/s13071-017-2330-2 (PMC5569544; doi:10.1186/s13071-017-2330-2)
Supplement: Supplementary file 2 — Results of all dog positive samples. (PDF 84 kb) [file 13071_2017_2330_MOESM2_ESM.pdf]

Table S1 Results of all dog positive samples

Province	Sample no.	Blood samples		Faecal samples	
		**LAMP**	**qPCR**	**LAMP**	**qPCR**
Lopburi	33	Positive	Positive (Cq 30.61)	ND	ND
Nakhon Pathom	6	Positive	Positive (Cq 28.43)	ND	ND
Nakhon Pathom	8	Positive	Positive (Cq 31.87)	ND	ND
Sa Kaeo	3	Negative	Negative	Positive	Negative
Sa Kaeo	5	Negative	Negative	Positive	Negative
Sa Kaeo	6	Negative	Negative	Positive	Negative
Sa Kaeo	7	Negative	Negative	Positive	Negative
Sa Kaeo	40	Positive	Positive (Cq 30.29)	Negative	Negative
Sa Kaeo	46	Positive	Positive (Cq 30.67)	ND	ND
Sa Kaeo	53	Positive	Positive (Cq 27.66)	ND	ND
Ratchaburi	25	Positive	Positive (Cq 29.87)	ND	ND
Ratchaburi	29	Positive	Positive (Cq 28.35)	ND	ND
Ratchaburi	41	Positive	Positive (Cq 32.07)	ND	ND
Ratchaburi	42	Positive	Positive (Cq 30.05)	ND	ND
Ratchaburi	48	Positive	Positive (Cq 29.77)	ND	ND
Ratchaburi	91	Positive	Positive (Cq 27.86)	ND	ND
Ratchaburi	94	Positive	Positive (Cq 30.92)	ND	ND
Ratchaburi	99	Positive	Positive (Cq 25.51)	Positive	Positive (Cq 31.25)
Ratchaburi	101	Negative	Negative	Positive	Positive (Cq 31.38)
Ratchaburi	102	Negative	Negative	Positive	Positive (Cq 32.85)
Ratchaburi	107	Negative	Negative	Positive	Positive (Cq 31.67)
Ratchaburi	111	Negative	Negative	Positive	Positive (Cq 32.11)
Ratchaburi	115	Positive	Positive (Cq 26.96)	ND	ND

ND = not determined

Cq = quantification cycle
